# Neuroinflammation in Prion Disease

**DOI:** 10.3390/ijms22042196

**Published:** 2021-02-23

**Authors:** Bei Li, Meiling Chen, Caihong Zhu

**Affiliations:** School of Basic Medical Sciences, Fudan University, Dong’An Rd 130, Shanghai 200032, China; Beili@fudan.edu.cn (B.L.); 19211010007@fudan.edu.cn (M.C.)

**Keywords:** neuroinflammation, prion disease, microglial activation, astrogliosis, neurodegenerative disease

## Abstract

Neuroinflammation, typically manifest as microglial activation and astrogliosis accompanied by transcriptomic alterations, represents a common hallmark of various neurodegenerative conditions including prion diseases. Microglia play an overall neuroprotective role in prion disease, whereas reactive astrocytes with aberrant phenotypes propagate prions and contribute to prion-induced neurodegeneration. The existence of heterogeneous subpopulations and dual functions of microglia and astrocytes in prion disease make them potential targets for therapeutic intervention. A variety of neuroinflammation-related molecules are involved in prion pathogenesis. Therapeutics targeting neuroinflammation represents a novel approach to combat prion disease. Deciphering neuroinflammation in prion disease will deepen our understanding of pathogenesis of other neurodegenerative disorders.

## 1. Introduction

Prion diseases, also called transmissible spongiform encephalopathies (TSE), are rare and fatal neurodegenerative disorders affecting both human and animals [1]. Prion diseases include Creutzfeldt–Jacob disease (CJD), Gerstmann–Sträussler–Scheinker syndrome (GSS), fatal familial insomnia (FFI), and kuru in humans, scrapie in sheep and goats, bovine spongiform encephalopathy (BSE, also known as mad cow disease) in cattle, chronic wasting disease (CWD) in cervids, transmissible mink encephalopathy (TME) in minks, feline spongiform encephalopathy (FSE) in felines, and the recently identified camel prion disease (CPD) in dromedary camels [2]. Additionally, prion diseases can be experimentally recapitulated in laboratory animals such as mice, hamster, and bank voles.

The term “prion” was coined by Stanley Prusiner in 1982 to describe proteinaceous infectious particles that account for the transmission of scrapie between sheep or goats [3]. According to the “protein-only” hypothesis first proposed by John Griffith in 1967 [4], prions are devoid of nucleic acid and mainly composed of scrapie prion protein (PrP^Sc^), a misfolded isoform of the host-derived cellular prion protein (PrP^C^). The gene-encoding prion protein (*Prnp*) was thereafter identified in hamster, mouse, and other mammals [5,6,7]. PrP^Sc^ and PrP^C^ share the same amino acid sequence. Posttranslational chemical modifications have not been found; however, there are significant differences in conformation. PrP^C^ contains abundant α-helix and minimal β-sheet, whereas PrP^Sc^ is predominantly composed of β-sheet. Nuclear magnetic resonance (NMR) analysis of recombinant PrP^C^ revealed an unstructured N-terminal flexible tail and a globular C-terminal domain comprising two-stranded antiparallel β-sheets and three α-helixes [8,9]. The high-resolution structure of PrP^Sc^ is still lacking largely due to its insolubility and heterogeneity of the purified infectious materials [10]. Nevertheless, it is now well accepted that the conformational transition from PrP^C^ to PrP^Sc^ represents a fundamental event during prion infection and propagation. As the principal component of prion, PrP^Sc^ acts as a template to recruit and convert PrP^C^ into nascent PrP^Sc^ with the same conformation, followed by fragmentation of the PrP^Sc^ aggregates to accrete further PrP^C^, thereby replicating and propagating the prion. The conformational differences of PrP^Sc^ determine diverse prion strains, which exhibit distinct disease phenotypes, such as clinical manifestation, incubation time, and lesion patterns, when transmitted to identical hosts. Different hosts also show varied susceptibility to various strains, which is known as the species barrier. The underlying factors discriminating prion strains may include glycosylation and sialylation of PrP^Sc^.

The etiology of prion diseases can be sporadic, inherited, or acquired by iatrogenic or dietary exposure to prions [11]. Although diverse etiologies differ in the initiator of disease cascade, they share a common fundamental molecular event and similar neuropathological features, which encompass spongiform changes, neuronal loss, and neuroinflammatory responses such as astrogliosis and microglial activation as well as the accumulation and deposition of PrP^Sc^ aggregates [11]. Here, we review the recent advances on neuroinflammation in prion disease.

## 2. Neuroinflammation in Prion Disease

The CNS has long been considered immune privileged, as the brain fails to mount an immune response to allogeneic grafts. But now it is well appreciated that the CNS is immune competent and does display features of inflammation [12]. The term “neuroinflammation”, meaning inflammation of the nervous system, was originally designated to describe immune responses (lymphocytic infiltrates, antibody production, and cytokine secretion, etc.) to infections, brain injury, toxic metabolites, or autoimmune diseases. More recently, however, the concept of neuroinflammation has been expanded to comprise chronic and sustained glial activation that is typically associated with aging and a variety of neurodegenerative diseases such as Alzheimer’s disease (AD), Parkinson’s disease (PD), frontotemporal dementia (FTD), and amyotrophic lateral sclerosis (ALS), as well as prion disease [13]. The involvement of neuroinflammation in neurodegeneration is a subject of intensive study. Accumulating evidence supports that neuroinflammation mediated by microglia and astrocytes may play an important role in the pathophysiology of neurodegenerative diseases [14]. Recently, a tsunami of genome-wide association studies (GWAS) has identified more than 100 gene variants associated with increased risk for developing AD, PD, FLD, or ALS. Many of the gene variants are related to immune response and highly expressed by microglia, highlighting the critical role of neuroinflammatory processes in neurodegenerative disorders [15]. 

Typically, prion infection does not elicit a prominent adaptive immune response in the periphery, probably due to self-tolerance associated with the similar immunogenicity of PrP^Sc^ with PrP^C^, which is constitutively expressed by the host [16]. Lymphocytic infiltration and the related inflammatory response are usually undetectable in prion-infected brains [17]. However, activation and proliferation of microglia are especially rampant in prion-infected brains. By immunohistochemical analysis, microglia have been observed to be closely associated with plaques in CJD, GSS, and kuru [18,19,20,21]. Compared to non-prion controls, the number of microglia is markedly increased in CJD and animal models of prion infection [22,23]. The morphology of the microglia also changes to an activated phenotype, suggesting a microglial response in prion diseases. Time course studies on experimental animals have found that microglial activation occurs at an early phase of the disease, preceding neuronal death, spongiform changes, and the onset of clinical symptoms [24,25,26]. Microarray analysis of prion-infected mouse brains has identified a unique expression profile of microglia [27]. Transcriptome profiling of mouse brains revealed that the genes upregulated by prion infection, irrespective of the prion strain or mouse genetic background, are expressed mainly by activated microglia [28], underscoring the central role of microglia in prion disease. These observations collectively imply that microglial activation actively drive prion-induced neurodegeneration rather than occurring merely as a secondary effect. However, the molecular mechanisms by which prion infections activate microglia and the functions of microglia in prion pathogenesis under distinct conditions remain largely unknown.

Astrogliosis has also been well recognized and documented in prion disease. Astrocytes, together with neurons, are considered important sites of prion replication. Early studies have shown that PrP^Sc^ deposit in astrocytes of both prion-diseased humans and rodents [29,30,31,32,33]. Astrocyte-specific expression of PrP^C^ could restore the susceptibility of *Prnp* knockout mice to prion infection, associated with PrP^Sc^ accumulation in astrocytes [34]. Primary astrocytes isolated from ovine PrP-overexpressing transgenic mice are capable of sustaining efficient replication and propagation of natural sheep scrapie [35]. Recently, human-induced pluripotent stem cell (iPSC)-derived astrocytes have been successfully established to efficiently replicate human prions in a cell culture model [36]. An immortalized mouse astrocyte cell line could selectively and differentially propagate different murine prions [37]. Taken together, these results suggest that astrocytes play a critical role in prion propagation and pathogenesis. Nevertheless, how prion infection trigger astrogliosis and the role of reactive astrocytes in prion disease are still undeciphered.

More recently, RNA-sequencing of prion-infected mouse brains collected at different time points found that conspicuous molecular changes can be detected at 8 weeks post inoculation, much earlier than previously anticipated and months before mice display clinical symptoms or brain damage. Interestingly, glial-related gene expression changes correlate precisely with the appearance of clinical signs, suggesting that glial perturbation, rather than the neuronal demise, could be the driver of disease [38]. Another longitudinal study comparing the transcriptome of prion-infected and uninfected mice by RNA-sequencing revealed unique molecular signatures of both microglia and astrocytes, which differ from the expression profiles of other neurodegenerative diseases [39]. Cell type-specific and genome-wide ribosome profiling of prion-infected mouse brains revealed that terminally sick mice with severe neurological symptoms displayed extensive molecular alterations in microglia and astrocytes, whereas only minor changes were detected in translational profiles of neurons. These results again imply that aberrant phenotype of glia suffice to cause disease and may even be the primary driver of prion-induced neurodegeneration [40].

Taken together, neuroinflammation, typically manifest as microglial activation and astrogliosis, along with transcriptomic changes, represents a rapidly emerging field of prion research. With the advances in the study on the neuroinflammation of prion disease, we believe it will deepen our understanding and facilitate innovative therapeutic approaches to combat this disease. 

## 3. Microglial Activation in Prion Disease

Microglia, the main resident macrophages in the CNS, are highly dynamic and motile [41]. Microglia play pivotal roles in the early development of the brain and maintenance of brain homeostasis throughout life. Microglia actively and continuously survey the brain parenchyma to find, uptake, and remove apoptotic cells or cell debris. Notably, microglia are also considered the most elaborate sensors of danger signals in the brain. In response to neural injury, infection, or neurodegeneration, microglia become activated and change their cell morphology, accompanied by marked proliferation and alterations in their expression profile. The central role of microglia in various neurodegenerative diseases is highlighted by genetic studies showing that variants of several microglia-expressed genes are important risk factors [42]. Microglia can be both beneficial and detrimental. The exact role of microglia in neurodegeneration may depend on their activated status and microenvironment, which can turn microglia into either friends or foes under distinct conditions [13].

In prion-infected mice, microglia are observed encompassing PrP^Sc^ deposits, and intracellular PrP^Sc^ can be detected within microglia [18,19,20,43,44]. The presence of prion in motile microglia indicates that microglia might facilitate the dissemination and spread of prion within the brain [44]. Furthermore, activated microglia in prion-infected brains produce pro-inflammatory cytokines that may contribute to neuronal damage [45,46]. However, microglia can engulf and degrade prions, thereby decreasing the prion load in the CNS and delaying disease progression [47,48]. The expression of anti-proinflammatory cytokine by activated microglia could also dampen neuroinflammation and ameliorate the pathogenesis [49]. The potential dual role of microglia in prion pathogenesis has prompted scientists to dissect the association between microglia and prion disease.

An early study using the synthetic peptide PrP106–126 (corresponding to the amino acid 106–126 of human prion protein) to treat primary cell cultures demonstrated that microglia are necessary for PrP106–126-induced cytotoxicity in primary neurons, suggesting that microglia are a mediator of neurotoxicity [50]. However, PrP106–126 is fundamentally different from bona fide prion, since the peptide has never been detected in vivo and is not infectious per se, which raises serious concerns about the relevance and validity of the findings. Another study reported that the CSF1 receptor (CSF1R) inhibitor, GW2580, restrained microglial activation and proliferation in prion-infected mice and thereby attenuated neuronal damage and slowed down disease progression, indicating a deleterious effect of microglial activation in prion diseases [51]. However, a contradictory effect was observed in another study using a different CSF1R inhibitor, PLX5622 (Plexxicon) [52]. These discrepant results suggest that pharmacological manipulation of microglia by targeting CSF1R may not be specific and therefore caution is indicated when employed in therapy. Indeed, CSF1R inhibition by PLX5622 affects myeloid and lymphoid compartment in the periphery and causes long-term changes in circulating and tissue-resident macrophages [53]. Accordingly, the role of microglia in prion diseases remains contentious, largely because of the dearth of appropriate model systems.

In an ex vivo model, ganciclovir-mediated radical and specific ablation of microglia in cerebellar organotypic-cultured slices (COCS) prepared from tga20/CD11b-HSVTK (overexpressed PrP in neurons and specifically expressed thymidine kinase of herpes simplex virus CD11b-positive myeloid cells including microglia) transgenic mice resulted in augmented PrP^Sc^ accumulation and elevated prion infectivity titer, suggesting that microglia play a crucial role in prion clearance [47]. Notably, neuronal loss was also significantly enhanced and neuropathological changes were remarkably aggravated upon microglial depletion [48]. When the tga20/CD11b-HSVTK mice were inoculated with prions and implanted with osmotic mini pumps to deliver the ganciclovir directly into the brain, disease progression was significantly accelerated [48]. Additionally, in prion-infected *Il34*^−/−^ mice that contain fewer microglia due to impaired microglial development and maintenance [54], PrP^Sc^ deposition was enhanced and incubation time was shortened compared with wild-type littermates [48]. Therefore, these results collectively suggest that microglia, rather than mediating prion pathogenesis, are potent defenders of the brain against prion disease. The beneficial role of microglia in prion disease was later confirmed by another independent study using an orthogonal approach [52]. Interestingly, microglia depletion does not affect anti-PrP antibody-induced neurotoxicity [55], indicating that microglia exert neuroprotection by decreasing the prion deposition instead of by affecting downstream events shared by prion infection and anti-PrP antibody treatment [56].

**Figure 1:**
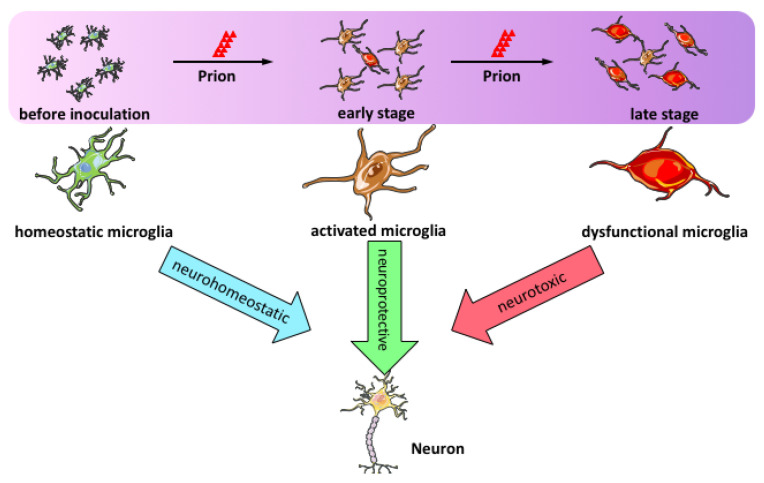
Microglial activation in prion disease. During prion disease progression, microglial activation is a complex and multistep process. Activated microglia consist of a heterogeneous population with distinct functions. At early stages, most microglia respond to prion infection and consequently enhance their phagocytic capacity to clear PrP^Sc^. Therefore, activated microglia at this stage are neuroprotective. However, the phagocytosis and clearance function are insufficient and sustained PrP^Sc^ accumulation causes neuronal damage, which could stimulate microglia to switch to a dysfunctional pro-inflammatory phenotype and elicit a detrimental effect in the brain.

A transcriptomic analysis of different brain regions with or without neurodegeneration revealed at least two types of microglial responses in prion-infected brains: A homeostatic response across all brain regions and an inflammatory response restricted to sites of neurodegeneration [57]. In concert with recent single-cell RNA-seq studies showing existence of heterogeneity and diversity of microglia subtypes in various mouse models of neurodegenerative diseases [58,59,60,61,62,63,64], we speculate that although microglia are generally neuroprotective, microglial activation during prion disease is a complex and multistep process. Activated microglia consist of a heterogeneous population with distinct functions. At early stages, most microglia respond to prion infection and consequently enhance their phagocytic capacity to clear PrP^Sc^. However, the phagocytosis and clearance function are insufficient, and sustained PrP^Sc^ accumulation causes neuronal damage, which could stimulate microglia to switch to a pro-inflammatory phenotype and elicit a detrimental effect in the brain (Figure 1).

## 4. Astrogliosis in Prion Disease

Astrocytes are the most abundant glial cells in the CNS that function in the maintenance of brain homeostasis and the blood–brain barrier, modulation of neurotransmission, energy supply, and many others [65]. Upon stimulation, astrocytes become activated and acquire reactive phenotypes manifest as morphological changes and upregulation of glial fibrillary acidic protein (GFAP) expression, a process called astrogliosis. Similar to activated microglia, reactive astrocytes can adopt both protective and harmful properties [66,67]. Astrogliosis represents another neuropathological hallmark of neurodegenerative diseases [68]. 

In prion disease, astrogliosis is pronounced and widespread as indicated by a significant increase in astrocyte cell numbers and associated GFAP signals. Interestingly, the response of astrocytes to prion infection is region dependent and heterogeneous. In a mouse model of prion infection, astrocytes in the hippocampus responded to PrP^Sc^ accumulation, whereas in the thalamus, the increase in GFAP and morphological changes were less pronounced despite stronger PrP^Sc^ deposition [69]. Therefore, prion infection-induced astrogliosis is context dependent and determined by the microenvironment of brain regions. With the disease progression, however, the region-specific homeostatic signature of astrocytes was partially lost and replaced by a uniform neuroinflammatory signature [70]. Recently, markers of reactive astrocytes have been applied to discriminate and stratify different prion strain or subtypes [71,72]. 

Recent studies suggest that reactive astrocytes are heterogeneous with different subtypes in distinct states and phenotypes [73,74,75,76]. Microglial activation and astrogliosis might be more closely related than previously assumed, as cytokine combination (TNFα, IL-1α, and C1qa) released by activated microglia could directly activate a subset of astrocytes into a neurotoxic phenotype (A1-astrocytes) [77]. In a transgenic mouse model of Parkinson’s disease, ablation of cytokines that stimulate A1 astrocytes results in neuroprotection [78]. To investigate the impact of A1-astrocytes on prion pathogenesis, TNFα, IL-1α, and C1qa triple-knockout mice were infected with prions. Although C3-positive A1-like astrocytes were significantly reduced, the amount of PrP^Sc^ deposition and titers of prion infectivity were not affected. Unexpectedly, triple-knockout even led to a significant acceleration of prion disease progression [79]. Given that lack of TNFα or C1qa has no effect on prion pathogenesis in the CNS [80,81], but single knockout of TNFα, IL-1α, or C1qa leads to decreased A1 astrocytes [77], the role of A1 reactive astrocytes in prion disease may be dose and context dependent. Interestingly, systemic inflammation induced by oral infection of a gastrointestinal helminth pathogen in prion-infected mice leads to earlier appearance of interferon γ receptor 1 (IFNGR1)-expressing reactive astrocytes and accelerated onset of prion disease [82]. Overall, these results suggest that reactive astrocytes in prion-infected mouse brains are more complex than the oversimplified A1 and A2 polarization, and their molecular features and functions in prion disease might differ from other neurodegenerative conditions.

Activation of unfolded protein response (UPR)-induced phosphorylated protein kinase R-like endoplasmic reticulum kinase (p-PERK) signaling has been observed in neurons in a wide spectrum of neurodegenerative disorders including prion disease [83]. Interestingly, p-PERK has also been detected in astrocytes of human tauopathy; however, the pathological contribution of astrocytic p-PERK signaling in neurodegenerative diseases remains unclear. A recent study found that dysregulated astrocytic p-PERK signaling turns astrocytes into distinct reactivity states, leading to a loss of synaptic trophism and neuroprotective functions. The specific block of astrocytic p-PERK signaling in prion-infected mice suffices to reverse this activation state and restores synaptic trophism and neuroprotective function, with improvement of behavioral deficits and increased survival [84]. Thus, targeting this astrocytic signaling represents a novel therapeutic approach for battling prion disease. These results also highlight the importance of astrocytes in prion pathogenesis. Indeed, astrocytes isolated from prion-infected mouse brain showed synaptotoxicity to primary cultured neurons, supporting the hypothesis that reactive astrocytes contribute to neurodegeneration in prion disease [85]. 

**Figure 2:**
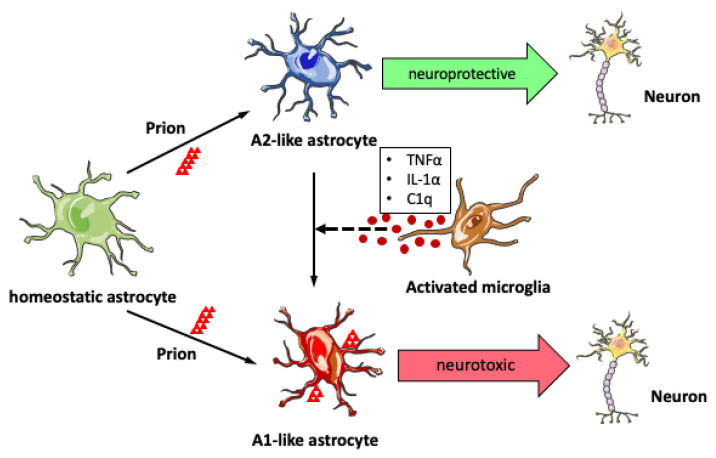
Astrogliosis in prion disease. Upon prion infection, astrocytes become activated into either the neuroprotective A2 phenotype or neurotoxic A1 phenotype. Heterogenous reactive astrocytes may coexist in prion-infected brains. Activated microglia release TNFα, IL-1α, and C1q factors which in turn could trigger the transition from A2 to A1 phenotype. Reactive astrocytes can replicate and accumulate prions, and their aberrant phenotype with dysregulated signaling plays an overall neurotoxic role in prion pathogenesis.

Taken together, reactive astrocytes not only replicate and accumulate prions in the brain, but their aberrant phenotype with dysregulated signaling also play an overall neurotoxic role in prion pathogenesis. Due to the existence of heterogeneous subtypes of astrocyte in prion-infected brains, further studies, such as single cell RNA-sequencing of astrocytes and cell-specific manipulation of certain astrocytic pathways, are required to dissect the precise role of each subpopulation in prion disease (Figure 2). 

## 5. Neuroinflammation-Related Molecules in Prion Diseases

### 5.1. Toll-Like Receptors

The innate immune receptor mediating microglial response to prion infection is not clearly defined. Toll-like receptors (TLRs) are a class of pattern-recognition receptors (PRRs) mainly expressed by innate immune cells, including microglia. TLRs sense and recognize a diverse range of invading pathogens to initiate the innate immune response. Therefore, it is conceivable that TLRs may mediate prion-induced microglial activation to defend against prion infection. 

Indeed, activation of innate immunity with a TLR ligand reduced prion accumulation, whereas suppression with a TLR inhibitor enhanced prion propagation in a mixed glial culture model [86]. Moreover, mice lacking TLR2 (C57BL/6, infected with 22L) or TLR4 (C3H/HeJ, infected with 139A or Me7) showed significant acceleration of prion progression, suggesting a protective function of TLR signaling in prion disease [87,88]. The contribution of TLR2 and TLR4 to prion pathogenesis seems to be Myd88-independent [89]. In agreement with these results, depletion of interferon regulatory factor 3 (*Irf3*^−/−^, C57BL/6J, infected with 22L, Fukuoka-1, or mBSE), a key transcription factor of the MyD88-independent type I IFN production pathway, resulted in a significantly earlier onset of prion pathology [90]. Interestingly, mice deficient in CD14 (C57BL/6), a glycosylphosphatidylinositol (GPI)-anchored protein that acts as a co-receptor of TLR4 to recognize pathogen-associated molecular patterns, survived longer than wild-type mice upon Chandler or Obihiro prion infection. This protective effect might be due to enhanced microglial activation and prion clearance, as well as elevated expression of anti-inflammatory cytokines [91,92].

Collectively, these results suggest an overall protective role of certain TLR in prion pathogenesis. But the full picture of various TLRs in prion disease is complex and context dependent. Further studies, such as cell type-specific manipulation of TLRs, are required to delineate the role of specific TLR signaling and downstream events within different cells in prion pathogenesis.

### 5.2. Complement System

In the CNS, components of the complement system are synthesized by microglia, astrocytes, and neurons [93]. In the CNS of prion disease patients and animal models of prion infections, early and terminal complement cascade components have been associated with PrP^Sc^ deposits [94,95,96,97]. Both classical and alternative pathways of the complement system were activated in prion-infected animals in a time-dependent pattern. Active complement compounds such as C1q, C3b, and membrane attack complex (MAC) were upregulated in various neural cells including microglia [96,97]. However, the contribution of complement activation to prion pathogenesis seems negligible, since mice lacking various complement components or their receptors succumbed to disease at a similar rate to the wild-type control upon intracerebral infection of prions [80,88,98,99]. Intriguingly, however, after peripheral challenge of prions, complement can trap and retain prions in secondary lymphoid organs, thereby facilitating peripheral prion replication and promoting prion pathogenesis [80,98,99,100,101,102].

### 5.3. Cytokines

Expression of pro-inflammatory cytokines, such as TNFα, IL-1α, and IL 1β, is significantly upregulated in the brains of both prion-infected animals and CJD patients [27,45,46,103,104]. Importantly, the temporal pattern of cytokine upregulation paralleled the onset and progression of molecular and clinical prion neuropathology [103]. Cytokines were predominantly induced in brain areas showing characteristic spongiform change and neuronal loss [104]. The spatiotemporal pattern suggests that cytokines may play a critical role in prion-induced neurodegeneration. Anti-inflammatory cytokine TGFβ was also upregulated in various animal models of prion diseases [105,106]. However, the temporal patterns and magnitude of upregulation varied substantially among models, differing with the prion strain and the species infected [105]. Similarly, expression levels of the anti-inflammatory cytokines such as IL-4 and IL-10 were also heightened in the cerebrospinal fluid (CSF) of CJD patients [107].

The contribution of cytokines to prion pathogenesis was examined. While most cytokines (TNFα, IL-6, TGFβ1, etc.) do not play a major role in prion-induced neurodegeneration, deficiency of IL-1 receptor 1 (IL-1R1, C57BL/6J, infected with 139A; B6;129S1/Sv, infected with RML), the receptor for IL-1α and IL-1β, caused a mild but statistically significant delay of disease progression [108,109], suggesting a detrimental role of IL-1R signaling in prion disease. Pathomechanically, depletion of IL-1R1 led to attenuated astrogliosis and enhanced microglial activation, resulting in augmented prion phagocytosis and clearance. These observations were supported by an association study that uncovered a correlation between SNPs in the *Il1r1* locus and incubation time of prion diseases [110]. In a mouse model of CJD, pharmacological inhibition of astrocytic IL-1R signaling could restore normal synaptic responses and reduce seizure susceptibility, suggesting that targeting IL-1 signaling may offer a novel symptomatic treatment for CJD [111].

Similarly, ablation of IL-4 or IL-13 had no overt effects on prion disease. However, knockout of IL-10 in 129Sv mice (infected with RML or ME7) markedly accelerated prion progression [112], suggesting that IL-10 exerts neuroprotective functions in prion pathogenesis. Interestingly, this effect seems to be dependent on genetic background, since another study observed only a modest acceleration of prion progression in *Il10*^−/−^ mice on a C57BL/6 background, but not on a 129S1/SvImj background (infected with RML) [109]. Therefore, both host genetic background and prion strain affect prion pathogenesis significantly. Additionally, constitutive knockout of certain gene may affect their functions in the periphery, such as in the gut, which could in turn influence the CNS via the gut–brain axis. Further studies, such as tissue- and cell type-specific conditional knockout, are required to dissect the precise role of specific cytokines in prion pathogenesis.

### 5.4. Chemokines

Expression of the CC chemokine ligand CCL2 is progressively upregulated in the CNS of C57BL/6 mice infected with ME7 prions, with upregulation temporally correlating with microglial activation. Upon inoculation of ME7 prions, CCL2 deficiency (C57BL/6) slightly prolonged the incubation time but had no impact on microglial activation or neuronal death [113]. Another study failed to find any alterations in disease progression after inoculation of RML prions into *Ccl2*^−/−^ mice (C57BL/6) [114], suggesting that the effect might be prion-strain dependent. Likewise, mice lacking the CCL2 receptor CCR2 (C57BL/6) experienced similar disease progression as wild-type mice following the RML prion challenge [17,109]. Expression levels of CCL5 and its receptors CCR1, CCR3, and CCR5 are also elevated in prion-infected mice [105,115]. Ablation of CCR1 (C57BL/6, infected with RML) led to a compensatory induction of CCL3 and CCR5, resulting in earlier activation of Erk1/2 in astrocytes and faster prion progression [116]. However, depletion of CCR5 had no effect on prion pathogenesis [109].

The levels of CXC chemokines ligands CXCL9 and CXCL10, which signal via receptor CXCR3, are upregulated by prion infection [108,117]. Interestingly, *Cxcr3*^−/−^ mice (C57BL/6) intracerebrally inoculated with 139A prion succumbed to disease with a delayed progression rate but accumulated more PrPSc in their brains [118]. Pathomechanically, knockout of CXCR3 decreased release of pro-inflammatory cytokines, which could account for the prolonged incubation time. On the other hand, ablation of CXCR3 blocked microglial activation and therefore impaired prion clearance, resulting in enhanced PrP^Sc^ deposition. The chemokine CXCL13 is also upregulated in prion disease [44,117]; however, deficiency of its receptor CXCR5 failed to alter prion pathogenesis after intracerebral challenge of RML prions [119].

CX3CR1, the receptor of a neuron-derived CX3C chemokine ligand CX3CL1 (also known as fractalkine), is primarily expressed by microglia in the CNS [120,121]. CX3CL1-CX3CR1 signaling represents an important neuron–microglia interaction that maintains microglia in a homeostatic phenotype [122]. The CX3CL1-CX3CR1 axis has been extensively studied in various models of neurodegeneration, yet conflicting results have been reported [123,124,125,126]. Altered CX3CL1-CX3CR1 signaling has been observed in animal models of prion infection [127,128]. *Cx3cr1*^−/−^ mice with Balb/c background infected with RML, Me7, and MRC2 prions experienced a slightly shortened incubation time compared to wild-type mice, suggesting a neuroprotective role of CX3CR1 signaling in prion disease [129]. Whereas *Cx3cr1*^−/−^ mice with a C57BL/6 background infected with RML and 22L failed to show any difference in disease onset, the duration and pathology was comparable to wild-type controls [130]. The dissimilar results may be due to the different mouse strains and genetic background and/or distinct experimental protocols used in each study. Notably, both studies observed no evidence of an impact of CX3CR1 deficiency on prion-induced microglial activation. Together, these studies suggest an overall weak effect of CX3CR1 signaling on prion pathogenesis.

### 5.5. Inflammation Regulators

Galectin-3, a regulator of inflammation, is mainly expressed by microglia in the brain. Upon prion infection, the galectin-3 level was significantly elevated [131]. Knockout of galectin-3 (C57BL/6, infected with 139A) slowed down prion progression without affecting PrP^Sc^ deposition or gliosis. However, lysosomal activation was profoundly suppressed by galectin-3 deficiency, suggesting that galectin-3 plays a detrimental role in prion disease by regulating lysosomal function [132].

Activation of the NLRP3 inflammasome has been observed in various neurodegenerative disorders and its contribution to pathology of these diseases has been studied [133,134,135,136,137,138,139]. In cell culture models, the NLRP3 inflammasome was found to be crucial for IL-1β secretion after treatment with PrP106–126 peptide [140] or PrP fibrils [141]. The role of the NLRP3 inflammasome in bona fide prion disease was assessed by intracerebral inoculation of *Nlrp3*^−/−^ and *Pycard*^−/−^ mice (C57BL/6) with RML prions. However, depletion of NLRP3 or ASC neither changed levels of IL 1β in the brain nor altered attack rates and survival times. Thus, NLRP3/ASC inflammasome does not play a major role in prion pathogenesis [142].

Immune checkpoints are regulators that protect immune system from attacking cells indiscriminately. The effect of blocking programmed cell death-1 (PD-1) pathway on mouse models of AD has been assessed, but conflicting results have been reported [143,144]. In prion disease, PD-1 expression was found to be restricted to microglia and highly increased following prion infection. Nevertheless, PD-1 deficiency (C57BL/6, infected with ME7) had no major impact on prion pathogenesis [145]. Similarly, lymphocyte activation gene 3 (Lag3, C57BL/6, infected with RML), an immune checkpoint receptor, was increased in prion infections, but did not overtly modify disease progression [146]. These results together suggest that blockade of immune checkpoint may not be an effective therapeutic strategy to halt prion diseases.

Sterile α and HEAT/armadillo motif-containing protein (SARM1), a member of the Myd88 family, negatively regulates innate immune response. Inactivation of SARM1 protects various forms of axonal degeneration [147,148,149,150,151,152]. Unexpectedly, upon prion infection, *Sarm1*^−/−^ mice (C57BL/6, infected with RML) displayed unaltered neuroinflammation but accelerated prion progression, ruling out an involvement of SAMR1-mediated immunity in prion pathogenesis. Transcriptomic analysis revealed that SARM1 deficiency upregulated expression of pro-apoptotic gene X-linked inhibitor of apoptosis (XIAP)-associated factor 1 (XAF1), thereby enhancing neuronal death and exacerbating disease [153]. Further study is required to delineate the crosstalk between SARM1 and XAF1, and their role in maintaining brain homeostasis.

### 5.6. Phagocytosis Mediators

The receptors and molecules that mediate the phagocytosis of prion remain to be elucidated. Milk fat globule epidermal growth factor 8 (MFGE8) is an opsonin that bridges apoptotic cells and phagocytes to facilitate phagocytosis by binding to phosphatidylserine on apoptotic cell membrane and integrins on phagocytes [154]. Because prion infectivity is often associated with lipids [155,156], it is conceivable that MFGE8 contribute to prion clearance by microglia. Indeed, mice deficient of MFGE8 (*Mfge8*^−/−^, infected with RML) displayed enhanced prion accumulation and experienced accelerated disease progression upon prion infection in a strain-dependent manner, since the effect of MFGE8 deficiency was observed in mice with C57BL/6 × 129Sv, but not with a C57BL/6 background [157]. The developmental endothelial locus-1 (Del-1, infected with RML), a homolog of MFGE8, does not complement MFGE8 for prion clearance in mice with a C57BL/6 background [158]. This finding suggests the existence of other unidentified polymorphic determinants of prion clearance.

The triggering receptor expressed on myeloid cells-2 (TREM2) is an innate immune cell receptor predominantly expressed by microglia in the brain. TREM2 facilitates engulfment and phagocytosis of apoptotic cells and bacteria, thereby quenching inflammation [159,160]. Dysfunctional variants of TREM2 have been identified as important genetic risk factors for developing AD and other neurodegenerative disorders [161,162,163,164,165]. Knockout of *Trem2* in a mouse model of AD (5xFAD) resulted in elevated hippocampal Aβ burden and augmented loss of cortical neurons, which are associated with markedly attenuated microglial activation [166]. Nonetheless, when *Trem2*^−/−^ mice (C57BL/6) were intracerebrally inoculated with RML prions, neither PrP^Sc^ deposition nor disease progression was altered, suggesting that TREM2 does not facilitate prion clearance. Interestingly, similar to what was observed in 5xFAD model, microglial activation was also diminished in prion-infected *Trem2*^−/−^ mice, indicating that TREM2 is involved in prion-induced microglial activation [167]. These results indicate that microglia-mediated phagocytosis and clearance of prion and Aβ may adopt distinct receptors and molecular pathways.

## 6. Therapeutics of Prion Diseases by Targeting Neuroinflammation

Prion diseases are so far incurable. Neuroinflammation offers as a new therapeutic target for prion diseases. Microglia play an overall neuroprotective role in prion pathogenesis, whereas aberrant phonotypes of reactive astrocytes may lead to neurodegeneration. Nevertheless, single-cell RNA-seq demonstrates that activated microglia and reactive astrocytes comprise heterogeneous subtypes and can adopt distinct phenotypes. While some subtypes are neuroprotective and beneficial, others could be more neurotoxic in the brain. Hence, it is conceivable to guide microglial activation and astrogliosis towards a beneficial phenotype that could efficiently clear prions, release anti-inflammatory and neurotrophic factors, and consequently slow down disease progression.

Early attempts to halt prion progression by blocking TLRs via administration of unmethylated CpG oligodinucleotides led to a delay in prion progression in mice peripherally inoculated with prions [168]. However, this effect may be neuroinflammation-unrelated and mainly due to alterations in the morphology and function of peripheral lymphoid elements [169]. CD14 deficiency confers neuroprotection from prion disease in mice by modifying cytokine release. Accordingly, the therapeutic strategy of using glimepiride to eliminate this GPI-anchored protein from the cell surface appears promising to modify prion progression [170]. Blockage of CSF1R signaling by GW2580 decreased microglial proliferation and the expression of pro-inflammatory cytokines in ME7-infected mice and improved behavioral performance and prolonged survival [51]. However, inhibition of CSF1R using PLX5622 (Plexxicon) impaired prion clearance and exacerbated prion pathology [52], suggesting that therapeutic strategies against prion disease should avoid simply eliminating or depleting microglia.

Microglia exert neuroprotection in the brain partly through engulfing and degrading prions. However, this process is inefficient, and animals eventually die [171]. Strategies aiming to strengthen phagocytic capacity of microglia have been explored. Retrovirus infection of prion-inoculated mice could transiently enhance microglial activation and prion clearance, although survival was not prolonged [172]. Intracerebral or systemic administration of lipopolysaccharide (LPS) to ME7-infected mice profoundly triggered microglial activation; however, this treatment exacerbated local inflammation and increased neuronal death [173].

Attempts to express anti-PrP scFv in brain-engraftable murine microglial cells followed by intracerebral transplantation into mice before or after prion infection have been reported. Intriguingly, injection of anti-PrP scFv-expressing microglia before or at an early stage of prion infection modestly delayed disease progression. This result supports a novel microglia-mediated immunotherapeutic approach to combat prion diseases [174].

## 7. Conclusion Remarks and Perspective

Neuroinflammation mediated by microglia activation and astrogliosis is now widely recognized as a hallmark of most types of neurodegeneration including prion diseases. While several models of microglial deficiency point to a neuroprotective role of microglia in prion pathogenesis, the exact molecular mechanisms underlying the microglial response to prion infection remains unclear. Emerging evidence suggests a crucial role of reactive astrocytes with aberrant phenotypes in prion-induced neurodegeneration. However, the underlying mechanisms of prion-induced astrogliosis are elusive and the precise involvement of reactive astrocytes in prion pathogenesis remains to be fully elucidated. Developing effective therapeutic strategies to modulate microglia and astrocytes in prion pathogenesis is challenging due to their heterogeneity and complex interactions with various cell types. Deciphering the orchestration of different subpopulations during prion progression represents a crucial step towards a better understanding of neuroinflammation in prion disease.

Recently, a study using single-nucleus RNA sequencing revealed that microglia and astrocytes in human and mouse respond differently to amyloidosis [175]. This cross-species difference may reflect the incomplete recapitulation of AD pathology in animal models. Since most studies of neuroinflammation in prion disease are based on animal models, whether microglial activation and astrogliosis in prion disease also differ between human and animal models remains to be determined.

A deeper understanding of neuroinflammation in prion disease may facilitate the development of techniques and approaches to modulate microglial activation and astrogliosis and thereby dampen disease progression. Since neuroinflammation is a common neuropathological hallmark of most neurodegenerative disorders, and prion disease shares many molecular and clinical similarities with other more prevalent neurodegenerative diseases such as AD and PD, we speculate the study of neuroinflammation in prion diseases could help uncover important insights into the role of neuroinflammation in other neurodegenerative conditions.
